# Understanding the Phase Behavior of a Multistimuli-Responsive Elastin-like Polymer: Insights from Dynamic Light Scattering Analysis

**DOI:** 10.1021/acs.jpcb.4c00070

**Published:** 2024-06-03

**Authors:** Peter C. Swanson, Galen P. Arnold, Carolyn E. Curley, Savannah C. Wakita, Jeffery D. V. Waters, Eva Rose M. Balog

**Affiliations:** School of Mathematical and Physical Sciences, University of New England, Biddeford, Maine 04005, United States

## Abstract

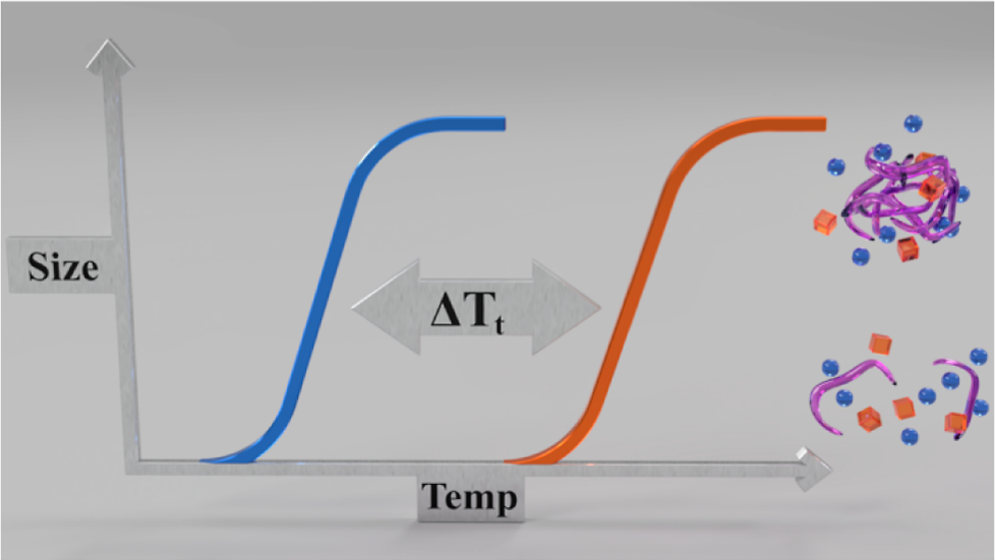

Elastin-like polymers are a class of stimuli-responsive protein polymers that hold immense promise in applications such as drug delivery, hydrogels, and biosensors. Yet, understanding the intricate interplay of factors influencing their stimuli-responsive behavior remains a challenging frontier. Using temperature-controlled dynamic light scattering and zeta potential measurements, we investigate the interactions between buffer, pH, salt, water, and protein using an elastin-like polymer containing ionizable lysine residues. We observed the elevation of transition temperature in the presence of the common buffering agent HEPES at low concentrations, suggesting a “salting-in” effect of HEPES as a cosolute through weak association with the protein. Our findings motivate a more comprehensive investigation of the influence of buffer and other cosolute molecules on elastin-like polymer behavior.

## Introduction

Genetically encoded protein polymers expand the scope of polymer science by harnessing the precision of recombinant protein engineering. By manipulating their amino acid sequence, protein polymers can be engineered to achieve specific physicochemical properties. One class of protein polymers, elastin-like polymers (ELPs), has been extensively studied owing to their tunable stimuli-responsive properties. ELPs are monodisperse, biocompatible, and amenable to chemical modification and processing, allowing for the creation of various architectures such as nano- and microparticles, films, and hydrogels.^1−6^ As such, they hold great potential in advanced functional materials, especially in biological applications such as drug delivery and scaffolds for tissue engineering. However, to be useful in real or engineered physiological settings, ELPs must be reliably stable and soluble. Furthermore, they must exhibit predictable stimuli-responsive behavior in a complex and dynamic solution environment. For example, for an ELP-based biosensor to function in biologically relevant conditions, it should ideally respond to a single stimulus, such as binding to a target biomolecule, while remaining insensitive to variations in local pH, electric potential, ion concentration, and the presence of other cosolutes, at least for the duration and conditions of the experiment. In bulk solution, the stimuli-responsive behavior of ELPs is characterized by a reversible transition between soluble, extended nanoscale polypeptide chains and micron-scale coacervates formed when the hydrophobic regions of the ELP seek burial through intra- and intermolecular association.^7−9^ This phenomenon, which has been referred to as coacervation, liquid–liquid phase separation (LLPS), aqueous two-phase systems (ATPS), and lower-critical solution temperature (LCST) behavior, has been exploited to create various architectures and assemblies.^10−14^ When coacervate assembly is not possible or favorable, such as when ELPs are end-tethered polymers to a solid substrate or in single-molecule simulations, single-chain ELPs will still undergo continuous collapse resembling a coil-to-globule transition, accompanied by changes to internal degrees of freedom, hydration, and stiffness.^15−17^

On the one hand, the LCST behavior of ELPs has proven to be highly modular; fusion or conjugation with an ELP domain marries stimuli-responsiveness to a wide variety of other functional molecules. On the other hand, the LCST behavior of the ELP is known to shift based on its fusion partner. For example, when using the LCST behavior of an ELP fusion as a nonchromatographic purification strategy, different ELP tags may be needed based on the solubility behavior of the desired tagged protein.^18^ Currently, there are valuable models for how ELP LCST behavior is tuned by the sequence length and composition of the ELP, including pH-sensitive ionizable residues, the solvent-exposed surface area of its fusion partners, and different ions in the Hofmeister series at different concentrations.^19−25^ Despite this body of work, we have found that it remains challenging to predict the LCST behavior of a given ELP in a specific set of conditions, including ions and cosolutes. Moreover, there is ongoing interest in understanding the contributions of colligative and intensive solution properties within the context of ELP LCST behavior.^26^ This work is fundamentally motivated by the potential to develop ELP-based biosensing systems capable of detecting specific biomolecules in complex, dynamic media through analyte-binding-induced changes in ELP conformational states. The engineering principles to develop such robust analyte-responsive polymers have not yet been defined.

Here, we provide a detailed description of the phase behavior of a polyelectrolytic ELP in different solution conditions and strive to interpret the results through a post-reductionist lens.^27,28^ This perspective acknowledges the full complexity of weak surface interactions, spatiotemporal partitioning, and solution environment in examining protein function.^29,30^ Dynamic light scattering (DLS) serves as our primary technique, surpassing turbidity measurements to offer valuable physical insights into protein dynamics, assembly, and microscale biomolecular condensation.^31^ The intricate interplay of influences on ELP LCST behavior is ultimately distilled in the rapid and accurate quantification of microscopic diffusion. In addition to offering new avenues for inquiry stemming from our results, we aim to provide an exemplar of responsible interpretation of DLS in the study of ELPs and similar systems.

## Experimental Methods

### Plasmid Construction

Synthetic genes encoding ELPs were purchased from Genscript (Piscataway, NJ, USA) and delivered in pUC57 vectors. Full DNA and protein sequences are provided in the Supporting Information (Table S1). Plasmids were subcloned using BsshII and NheI restriction enzymes and standard molecular biology techniques into a custom vector referred to as POE-W. The POE-W vector appends a single tryptophan residue at the C-terminus of the expressed protein, as well as an N-terminal pelB leader peptide that facilitates the translocation of the expressed protein to the periplasm and is cleaved upon export. Validity of the signal peptide cleavage site was confirmed via the SignalP Web server (Figure S1). Plasmids were transformed using conventional heat shock methods into NEB5α chemicompetent cells (New England Biolabs, Ipswich, MA, USA) and purified using NEB Monarch Plasmid Miniprep Kits. DNA concentration was measured with a ThermoFisher NanoDrop 2000c UV–vis spectrophotometer. Plasmid construction was confirmed by Sanger sequencing (Eurofins Genomics, Louisville, KY, USA).

### Protein Expression and Purification

Chemically competent BL21(DE3) *Escherichia coli* (New England Biolabs, Ipswich, MA, USA) were transformed with the POE-W ELP expression plasmids and plated on 2XYT agar +100 μg/mL carbenicillin solid medium. Fresh transformants were picked with a sterile loop and used to inoculate 15 mL starter cultures of 2XYT or SuperBroth +100 μg/mL carbenicillin. Starter cultures were grown at 37 °C shaking at 200 rpm until visibly cloudy, typically 2–4 h. The full volume of culture was then used to inoculate 1 L of freshly autoclaved 2XYT or SuperBroth + carbenicillin. Liter cultures were returned to shaking at 37 °C for 24 h following inoculation. The leaky T7 promoter resulted in high levels of recombinant protein production without induction. Cells were harvested by centrifugation (4200*g*, 4 °C, 20 min) and either processed for protein purification immediately or stored at −80 °C. Following periplasmic extraction, ELP was purified using an inverse transition cycling protocol adapted from Hassouneh et al.^18,32^ Following purification, ELP was dialyzed into deionized water, lyophilized, and stored at −20 °C. Purity was assessed using SDS-PAGE, followed by Coomassie (KI8) or silver (I90) staining. Our full protocol for purification of ELPs from periplasmic expression systems is available on protocols.io.^32^ Full gene and protein sequence information is provided in the Supporting Information (Table S1).

### Dynamic Light Scattering

DLS and zeta potential measurements were performed on either a Malvern Zetasizer Nano ZS or a Malvern Zetasizer Ultra Red Label. Measurements were made at a scattering angle of 173° using a He–Ne laser with λ = 633 nm. Size-only measurements involved adding 100 μL of sample into single-use low-volume cuvettes, while zeta measurements were performed with Malvern folded capillary zeta cuvettes, where cuvettes were filled to the desired volume specified on the cell. For sample preparation, lyophilized protein was dissolved in a prechilled solvent on ice for a duration of 10 min, accompanied by intermittent vortexing to ensure thorough dissolution. To establish the absence of any potential contamination, solvent scattering measurements were conducted prior to the ELP measurement. Solvents were prefiltered before ELP dissolution using a 0.4 μm syringe filter. ELP DLS measurements were conducted in triplicate for each specified temperature point. The temperature-adjusted viscosity of water was used as the solvent viscosity parameter. Samples equilibrated for 120 s between the incremental temperature shifts of 2 °C before size measurements; there was no equilibration time between the zeta potential and size measurements at a given temperature.

### Software, Data Analysis, and Statistics

Size distribution histograms were created from the DLS autocorrelation curves via a cumulant fit performed by ZS Xplorer v3.30. From the size distribution data, the mean intensity size value from the largest percent volume peak was reported as the hydrodynamic diameter. Microsoft Excel 2019 was used for data curation and storage, and MATLAB R2022a was used for data workup and creation of graphs. All data not contained within the paper or supporting files are available in the Dryad data repository with DOI 10.5061/dryad.wstqjq2tq.

## Results and Discussion

**Figure 1:**
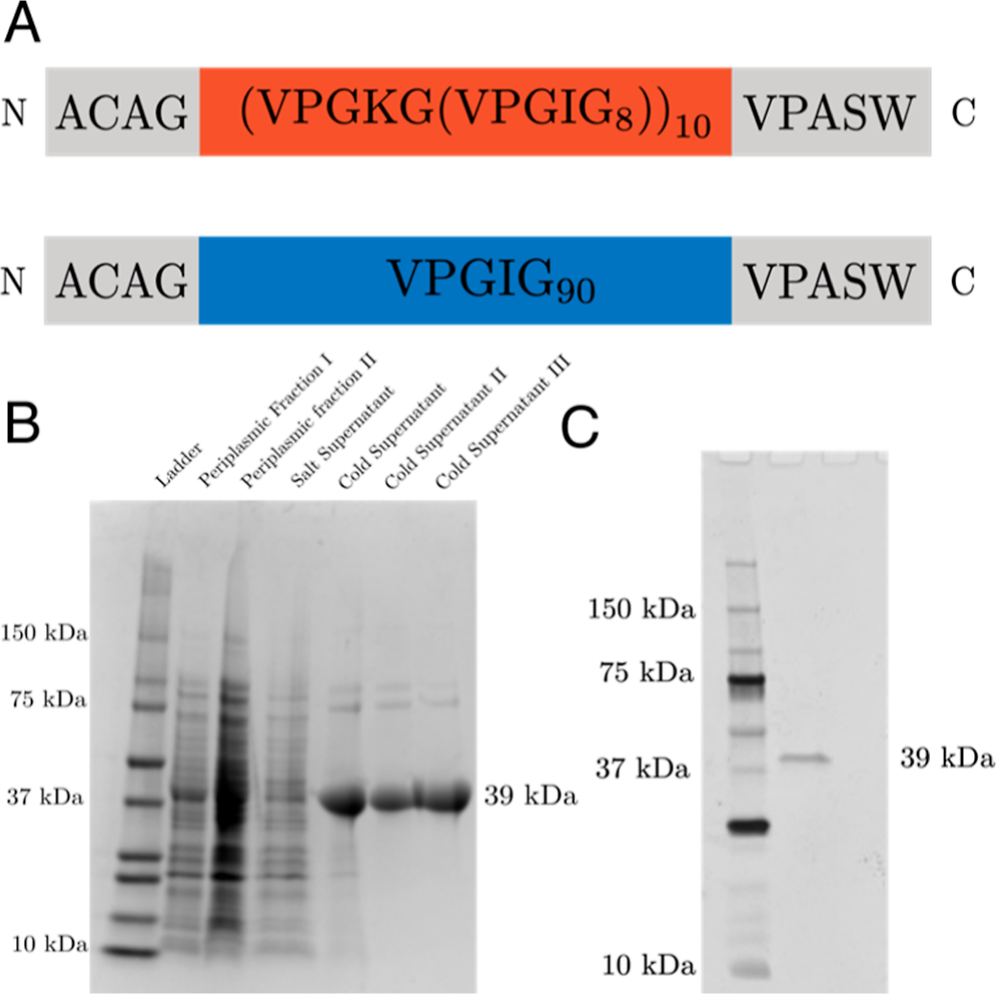
Design, expression, and purification of ELPs. (A) Amino acid sequences of the ELPs KI8 (top) and I90 (bottom). (B) SDS-PAGE of the purification of KI8 from the periplasm of *E. coli* using inverse temperature cycling. (C) SDS-PAGE of purified I90.

We designed, expressed, and purified an ELP that we refer to as KI8 because the body of the polymer consists of the amino acid sequence [(VPG**K**G)(VPG**I**G)_**8**_]_10_ (Figure 1a). Lysine’s ε-amino groups have a reference p*K*_a_ value of 10.54, although this value may shift depending on the specific microenvironment.^33^ The ionizable Lys residues were incorporated to confer pH responsiveness to KI8, which has a predicted pI = 10.6. We also generated an ELP we called I90, which is the same length as KI8 but consists of the amino acid sequence (VPGIG)_90_ to serve as a comparison polymer that lacks pH-responsiveness and the charge/polarity of the lysine side chains. KI8 and I90 were purified to homogeneity by inverse temperature cycling and characterized for purity using SDS-PAGE (Figure 1b).^18,32^ Note that because both polymers have a single Cys residue near the N-terminus, formation of dimers via disulfide bridges is possible.

**Figure 2:**
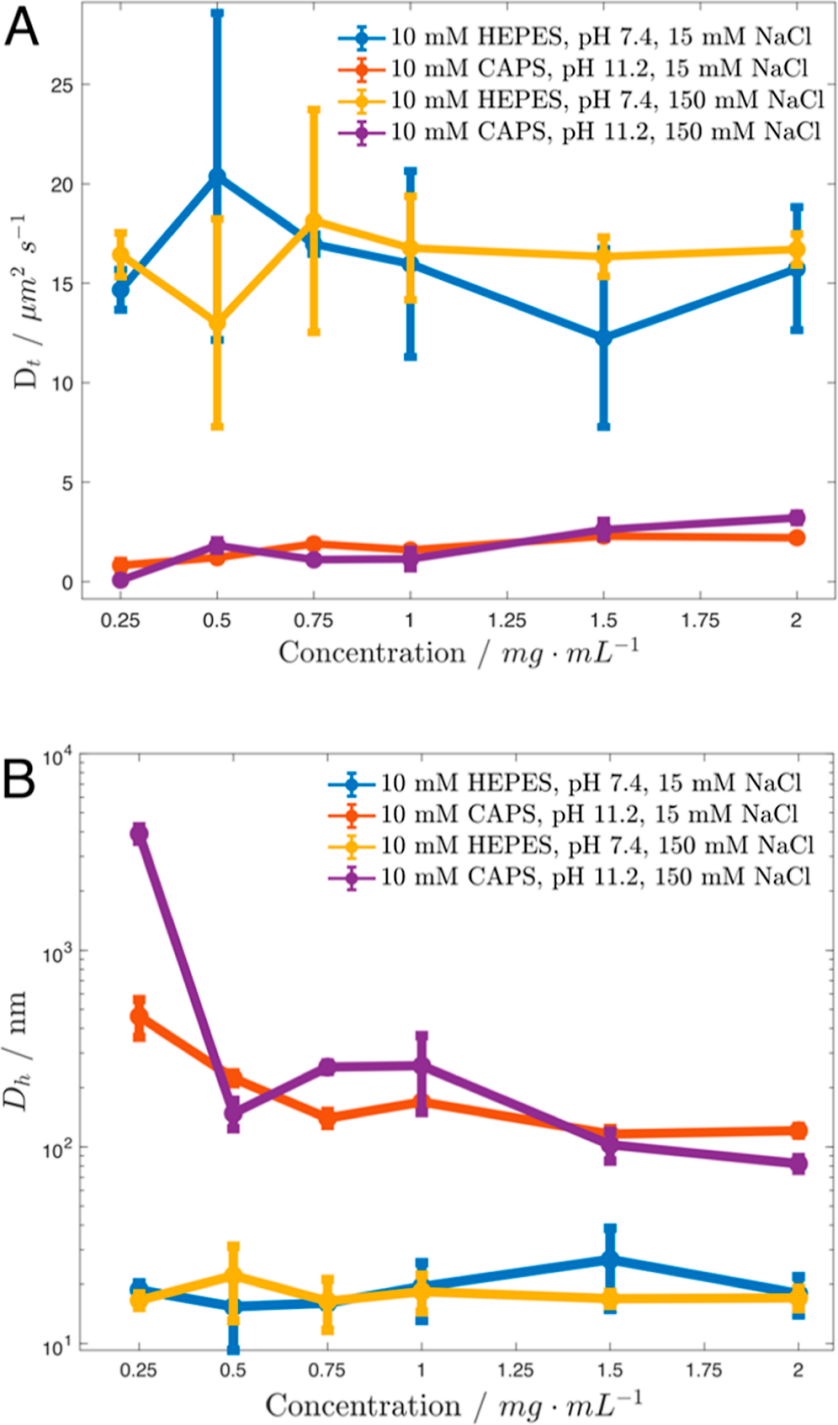
Concentration dependence of KI8 behavior. (A) Dependence of the translational diffusion coefficient on concentration in solutions of KI8. (B) Dependence of the calculated hydrodynamic diameter on concentration in solutions of KI8.

DLS theory for particle sizing relies on the infinite dilution assumption, which assumes that the scattering behavior of particles can be attributed to their independent, translational Brownian motion, not to attractive or repulsive intermolecular interference.^34^ This assumption is especially relevant for ELPs in general, and KI8 in particular, due to the tendency of ELPs to self-associate and the tendency of similarly charged proteins to experience repulsion. To determine the validity of the infinite dilution assumption for the system under study, we measured the concentration-dependence of the hydrodynamic diameter (*D*_H_) and translational diffusion coefficient (*D*_T_) of KI8. We performed DLS on serial dilutions of KI8 under four conditions of varying pH and salt (NaCl) concentration (Figure 2). We found *D*_H_ ≈ 15–26 nm in 10 mM HEPES pH 7.4, 15 mM or 150 mM NaCl, with no clear trend as a function of concentration. This size measurement likely corresponds to dynamic, soluble monomers of KI8, as it falls between the predicted size of a 39 kDa folded globular protein (∼2 nm) and the size of a fully extended beta strand conformation of 460 amino acids (161 nm). We also found that in 10 mM CAPS pH 11.2, 15, or 150 mM NaCl, the predominant species detected were greater than 100 nm, indicating the greater tendency of these samples to assemble. We hypothesize that this tendency to assemble persists even at lower concentrations because these samples were prepared by dilution of a stock initially dissolved at a concentration of 2 mg/mL, promoting self-assembly through concentration effects. Samples initially dissolved in these same conditions at 0.4 mg/mL were later seen to have nanometer-scale *D*_H_ values. We interpret the possible outliers at 0.25 mg/mL as potentially resulting from nearing the lower limit of the concentration range for accurate measurements. Even though we have confirmed that we are in a regime where the infinite dilution assumption is permitted, particle size distribution is still a derived metric that depends on variables such as solvent viscosity and refractive index and assumes homogeneous, hard spherical particles.^35^ Readers are advised to interpret hydrodynamic size values provided by DLS as hypothetical and useful for comparison only within a given experimental system.^36^

Because DLS measures particle motion, it offers insights into the molecular interaction parameters *B*_22_ and *k*_D_. The second virial coefficient *B*_22_ is a thermodynamic parameter that characterizes the attractive or repulsive interaction potential between neighboring particles. Positive *B*_22_ values indicate repulsive interactions, minimizing aggregation, while negative values of *B*_22_ indicate attractive interactions that promote aggregation. *B*_22_ can be qualitatively assessed by examining the concentration series depicted in Figure 2b. At pH 7.4, no size trend with respect to concentration is observed, suggesting no strong attraction or repulsion under these conditions. On the other hand, in the high pH samples, the observed trend of a decreasing hydrodynamic size with increasing concentration suggests the presence of repulsive forces, as it indicates an increase in diffusive motion. The DLS interaction parameter *k*_D_, which is derived from the slope of the plots in Figure 2a, is also called the diffusion virial coefficient.^37,38^*k*_D_ measures the dependence of diffusivity on concentration and takes into account not only thermodynamic effects captured by *B*_22_ but also electrostatics, excluded-volume contributions, and hydrodynamic friction due to steric hindrance. This can make physical interpretation of *k*_D_ values complex, as different contributions dominate under different solution conditions.^38^ Nevertheless, *k*_D_ is frequently used to assess aggregation behavior, where positive values suggest colloidal stability and negative values suggest a tendency for aggregation. Consistent with the qualitative *B*_22_ observations, *k*_D_ is positive in the samples at pH = 11.2 and lacking a clear trend in the samples at pH = 7.4. This is interesting because one would expect greater electrostatic repulsion in the lower pH samples in which KI8 is charged. These results may reflect greater stability gained upon partial aggregation of KI8 at high pH; however, electrostatic interactions and possible buffer specific effects may also be at play. For example, in both HEPES and CAPS buffers, *k*_D_ is reduced when NaCl concentration is increased from 15 mM to 150 mM. The influence of ionic strength on *k*_D_ has also been observed for bovine serum albumin in Tris, phosphate, and citrate buffers.^39^ This dependency has been attributed to reduced repulsive interactions due to electrostatic screening, the extent of which varies based on the buffer’s chemistry. We expect that this effect also manifests in ELPs; considering their distinctive, low-complexity composition and well-characterized stimuli-responsive properties, they present an intriguing system for further investigation.

**Figure 3:**
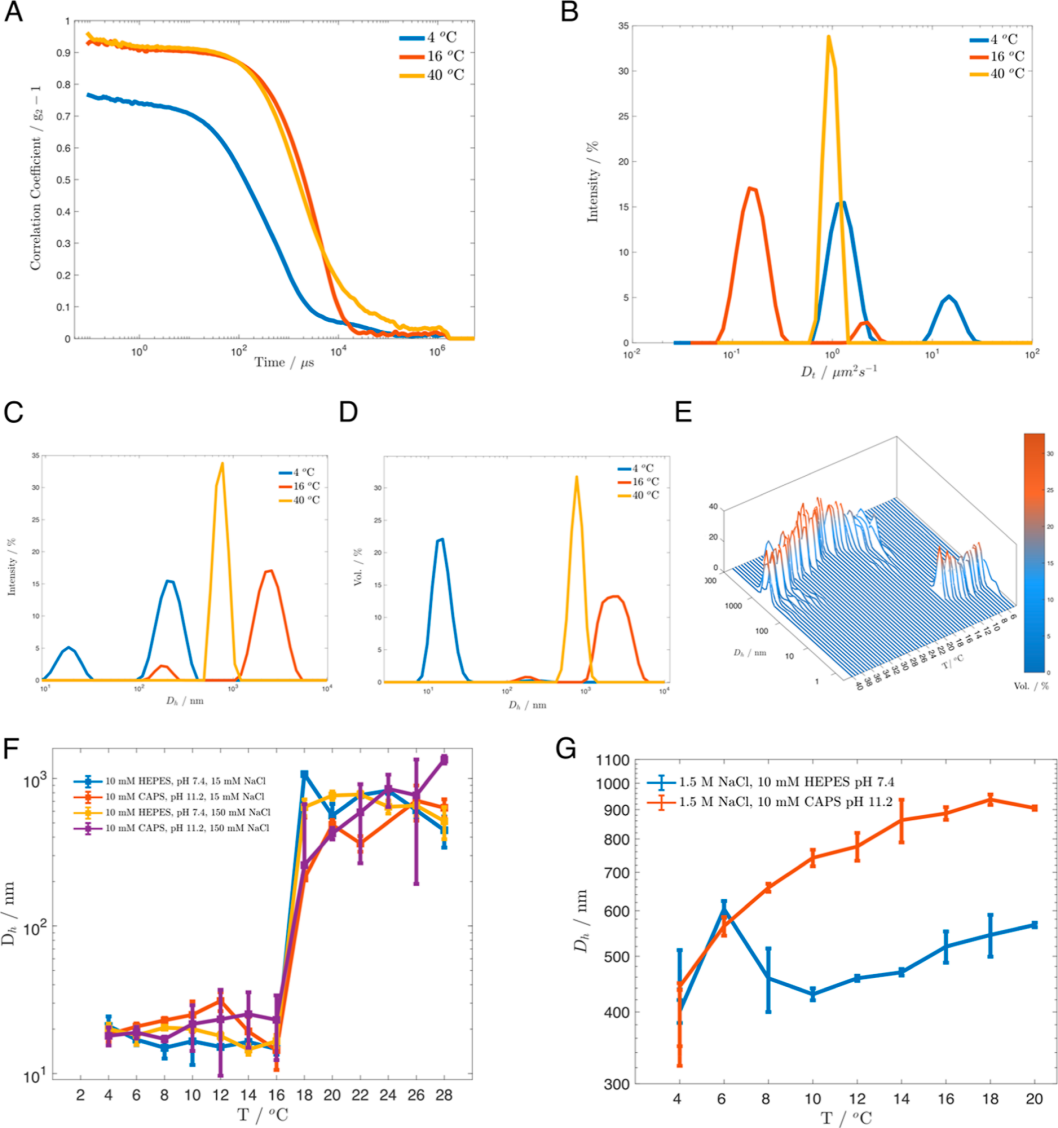
Temperature dependence of KI8 behavior. (A) Representative correlation functions for KI8. (B) Representative diffusion distributions for KI8. (C) Representative intensity-based particle size distributions for KI8. (D) Representative volume-based particle size distributions for KI8. (E) Volume percent peaks from a representative temperature ramp particle sizing DLS experiment. Panel A–F measurements were made with 0.4 mg/mL KI8 in 10 mM HEPES pH 7.4, 15 mM NaCl. (F) Determination of the transition temperature of KI8 in four different conditions. (G) Temperature-dependent behavior of KI8 assemblies in the presence of high salt concentration.

Previously, Conticello and colleagues characterized an elastin-mimetic protein of similar composition [(VPGVG)_4_(VPGKG)]_39_.^40−42^ This polymer, which has overall fewer repeats and a higher charge density than KI8, displayed a strong pH-dependence of transition temperature (*T*_t_).^42^ To investigate the pH- and ionic-strength dependence of the LCST of KI8, we performed temperature-dependent DLS from 4 to 40 °C in the same four different solution conditions used for the concentration series above, at a concentration of 0.4 mg/mL (10 μM) KI8. Figure 3 presents our data analysis workflow. The most basic graphical data provided by modern DLS instrumentation is the autocorrelation function; faster diffusing particles will decay to the baseline more rapidly (Figure 3a). A multiple exponential is then fit to the correlation function to obtain the intensity-weighted distribution of diffusion coefficients (Figure 3b). Particle size is then calculated from translational diffusion coefficients using the Stokes–Einstein equation (Figure 3c). Finally, for polymodal samples such as our ELPs, it is important to convert the intensity distribution to a volume distribution to correct for the greater scattering of larger particles (Figure 3d). This will provide a more realistic approximation of relative populations of different sizes. In all our analyses, we determined the majority peak based on the volume distribution, then assigned the corresponding size or diffusion coefficient value from the intensity distribution for greater accuracy. A 3D plot of volume distribution as a function of temperature shows the abrupt transition from nanometer-scale particles to micron-scale particles (Figure 3e). We predicted that at the lower pH, the greater charge on KI8 would increase its *T*_t_ compared to the lower pH at each salt concentration. Surprisingly, we observed no difference in *T*_t_ across these four conditions (Figure 3f). This contrasts with evidence of other basic ELPs with strongly pH-dependent transition temperatures in 50–140 mM NaCl.^21^ We hypothesize that this difference may be attributed to the lower linear charge density of KI8, leading to greater susceptibility to screening effects. We can also compare this result to a recent systematic study of acidic pH-responsive ELPs of varying length and composition.^14^ The authors of this study also find that some of their ELPs are pH-insensitive under certain conditions despite having many ionizable residues. The authors also found that higher [NaCl] did not always decrease *T*_t_, but in at least one case increased it, perhaps via a salting-in effect. This supports the conclusion that the effect of salt and pH on ELP solubility are highly sequence-dependent.

Given this result, we asked under what conditions any pH-dependent effects could be recovered. We observed that in the presence of 1.5 M NaCl, KI8 was coacervated at 4 °C (Figure 3g). This result is expected, as high concentrations of salt dramatically depress the transition temperature of ELPs. With increasing temperature, the hydrodynamic diameter of the phase-separated particles at pH = 11.2 continued to grow, while the size of the particles at pH = 7.4 remained constant. This is consistent with the hypothesis that at pH = 7.4, KI8 coacervate particles form in a manner that creates a positively charged surface area, resulting in mutual particle–polymer repulsion and thus an opposing force to assembly beyond the observed size plateau. At pH 11.2, a smaller proportion of Lys residues are positively charged, resulting in a lower repulsive force and a greater tendency to associate until an equilibrium is reached at a greater surface area.

**Figure 4:**
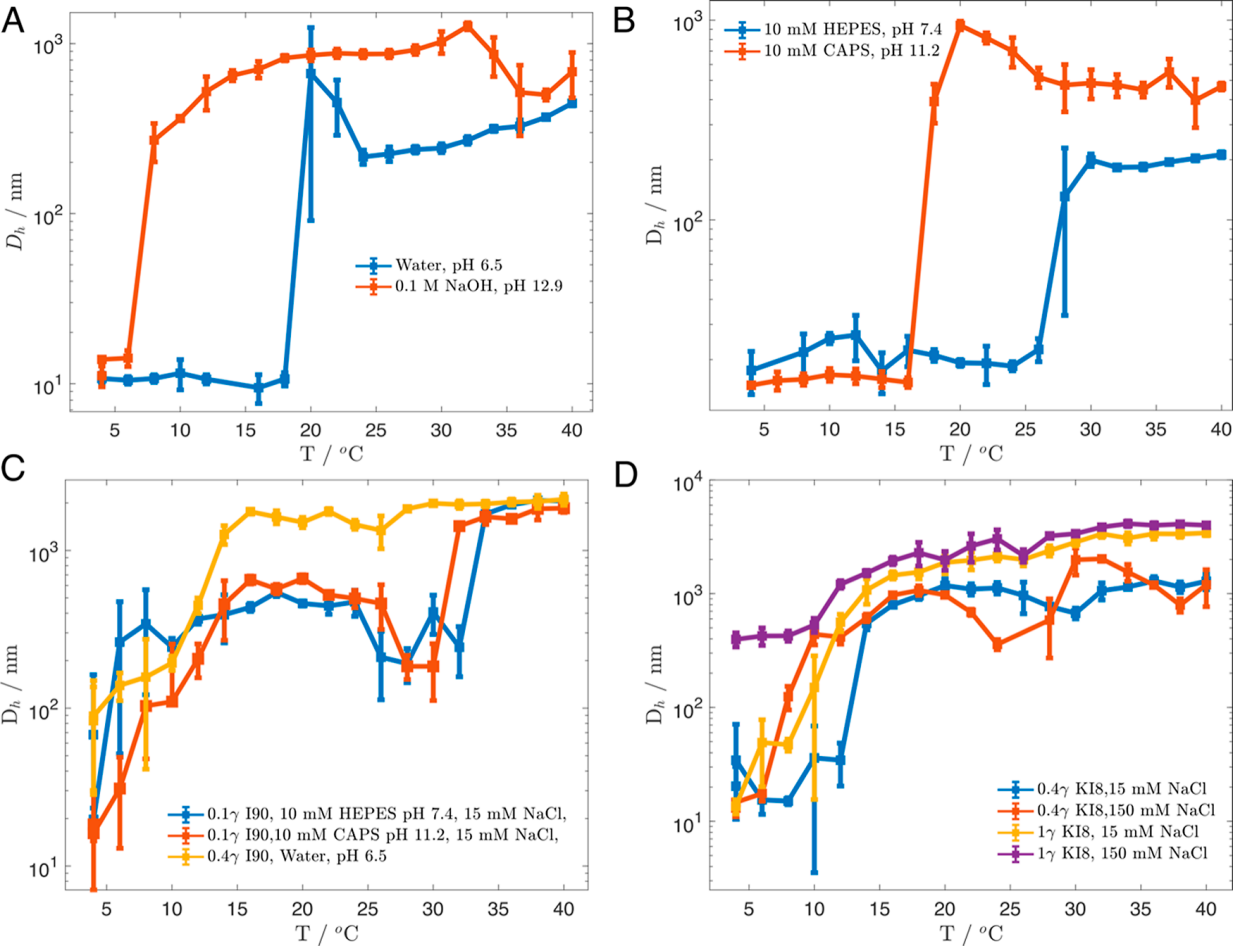
Independent effects of pH and salt on KI8 stimuli-response. (A) KI8 is highly responsive to NaOH-induced pH change. (B) In the absence of NaCl, KI8 displays a pH-dependent shift in transition temperature. (C) Uncharged polymer I90 is highly nonpolar and displays distinct stimuli-response behavior. (D) In the absence of buffer, KI8 is responsive to salt concentration.

We observed a pH-dependent shift of the *T*_t_ for KI8 from ∼20 °C in water (pH = 6.5) to 6 °C in 0.1 F NaOH (pH = 12.9) (Figure 4a). Furthermore, in buffered solution without NaCl (10 mM HEPES pH 7.4 vs 10 mM CAPS pH 11.2), we also observed a pH-dependent Δ*T*_t_ for KI8 of ∼8 °C (Figure 4b). Based on its theoretical p*K*_a_, the Lys residues of KI8 are expected to be mostly (82%) but not fully deprotonated at pH = 11.2, while at pH 12.9, they are expected to be almost completely (99.5%) deprotonated. The large decrease in *T*_t_ over this relatively small pH change may be due to an outsize contribution of a small amount of charge to the solubility of KI8, or it may be due to other solution differences irrespective of the extent of Lys protonation such as the presence of cosolute buffer molecules or Na^+^ ions. We also consider in our interpretations that the p*K*_a_ of Lys residues in KI8 may be decreased by the hydrophobicity of their environment within the ELP, and that the degree of hydrophobicity experienced by the Lys residues depends on the phase state.^43^

As a control for the contribution of Lys residues, we also investigated the LCST behavior of an ELP the same length as KI8 but consisting only of VPGIG repeats (“I90”, Figure 1a, bottom). At a concentration of 0.4 mg/mL in filtered, deionized water, the same concentration as the previous experiments performed with KI8, I90 was observed as monomer size (15–20 nm) at 4 °C (Figure 4c). With increasing temperature, I90 particles gradually increased in size to ∼500 nm, then jumped to ∼1200 nm at 14 °C and continued to grow to over 2 μm in hydrodynamic size. Given its low apparent *T*_t_, subsequent I90 experiments were performed at 0.1 mg/mL. Still, in both 10 mM HEPES pH 7.4, 15 mM NaCl and 10 mM CAPS pH 11.2, 15 mM NaCl conditions, I90 began to coacervate at 6–8 °C, continued to assemble to 200–500 nm particles, then jumped to 1.5–2 μm diameter particles above 30 °C. There are several comparisons one can make between KI8 and I90 LCST behaviors. First, both I90 in water and KI8 in 0.1 NaOH transition around 6–8 °C, with very similar temperature trend profiles. This supports the validity of considering KI8 at high pH to be fully deprotonated. However, in both buffered, low-salt conditions, I90 was much less soluble than KI8, even at a lower protein concentration. Second, I90 shows no difference in *T*_t_ with changing pH/buffer conditions. This implies that pH- and buffer-dependent effects observed in KI8 are dependent on the presence of Lys residues, and that a Cys residue and N- and C-termini are not sufficient to confer pH-responsiveness. This control is significant because high pH can promote disulfide bond formation, which would typically decrease *T*_t_ by increasing polymer size through disulfide-mediated dimerization. If this effect were present, it should be observed in both I90 and KI8. However, I90 coacervation began at 6–8 °C under both neutral and high pH conditions, indicating that pH-mediated disulfide bond formation is not a significant factor for the observed buffer-dependent effects.

We also asked under what conditions a salt-dependent shift in *T*_t_ could be observed for KI8. The transition temperature of ELPs has been shown previously to depend linearly on NaCl concentration.^23,44^ In our initial experiments in buffered conditions, identical *T*_t_ were observed at 15 mM and 150 mM NaCl. However, in the absence of buffer, we observed a decrease in *T*_t_ with increasing NaCl as expected (Figure 4d). With increased KI8 concentration (1 mg/mL), the sensitivity of the *T*_t_ to salt concentration increased further. This is consistent with the hypothesis that salt is playing a dual role in lowering the *T*_t_ of KI8, both by increasing solvent polarity and reducing charge–charge repulsion between polymers by electrostatic screening. KI8 also had a lower *T*_t_ without buffer at both NaCl concentrations. Taken together with the I90 results, these results further provoked us to investigate the hypothesis that there may be buffer-specific interactions with KI8 contributing to its increased solubility even in the presence of NaCl.

**Figure 5:**
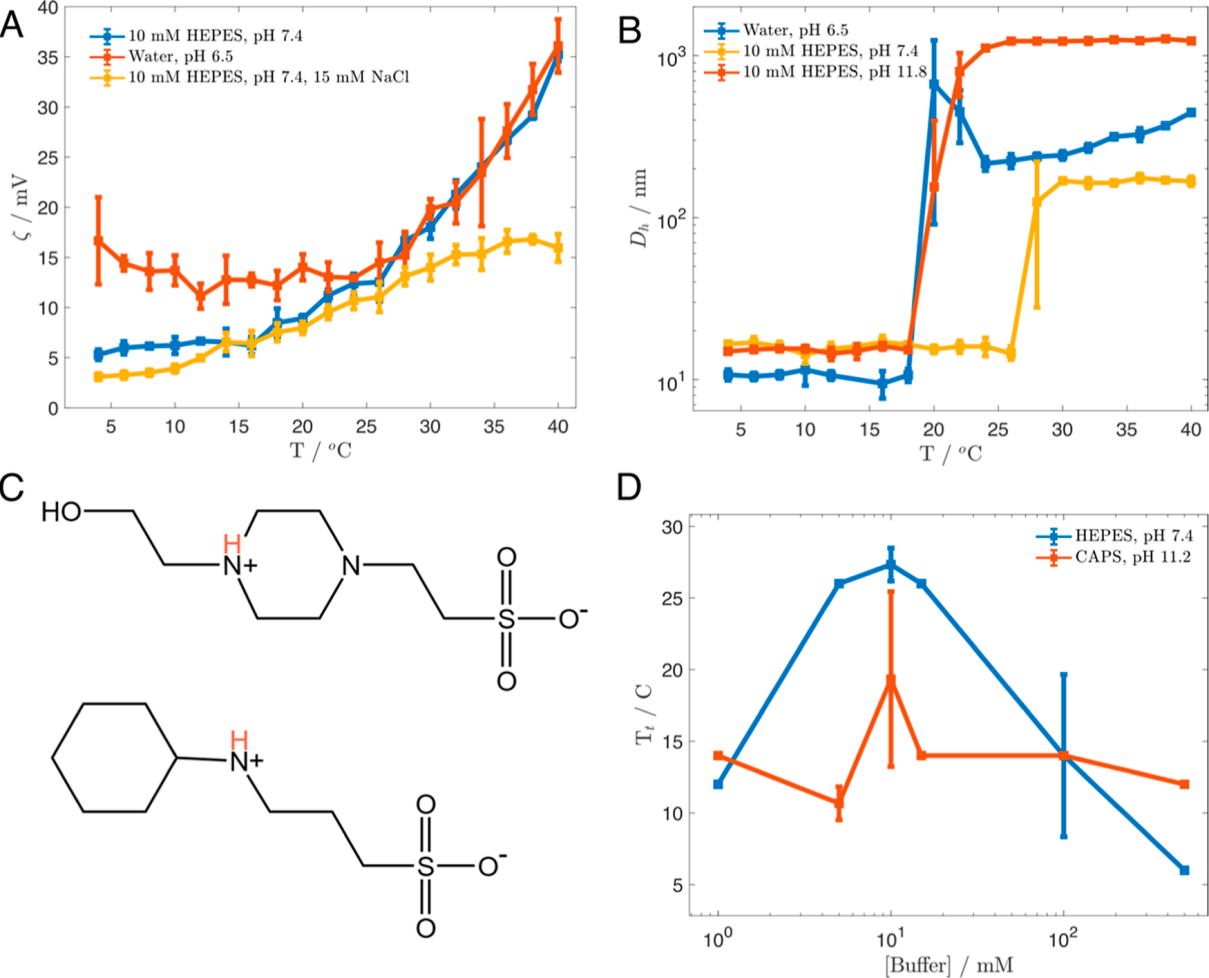
Effects of buffers on KI8 surface and stimuli-response. (A) Temperature-dependent zeta potential measurements of KI8. (B) Temperature response of KI8 in HEPES at neutral and high pH. (C) HEPES (top) and CAPS (bottom) molecules, with main ionizable proton shown in red. (D) Transition temperature of KI8 at various buffer concentrations.

Previous studies have shown that HEPES can adsorb onto protein surfaces, influencing phase separation behavior by inducing a “salting-in” effect through weakly attractive, exothermic interactions between HEPES molecules and protein particles.^45−47^ Supporting the hypothesis that this phenomenon is occurring for KI8, we observed a reduction in surface charge for KI8 below the *T*_t_ in the presence of 10 mM HEPES as reflected by zeta potential measurements (Figure 5a). This result is consistent with the interpretation that HEPES buffer molecules adsorb onto individual KI8 monomers, modifying their electrophoretic mobility and thus their measured zeta potential. Buffer molecules have been observed to exert significant effects on zeta potential of proteins, including surface charge reversal, at low millimolar and even micromolar buffer strength.^48^ At these low concentrations, buffer specific effects have been attributed predominantly to dispersion forces acting at the protein surface, rather than classical Hofmeister phenomena operating at higher concentrations to influence water structure.^49^ We hypothesize that when protein–protein interactions become stronger than protein–buffer interactions (i.e., above the *T*_t_), the zeta potential of the phase-separated KI8 particles now follows the same temperature trend as the water solution. In the presence of NaCl, a strong electrolyte, the surface charge on KI8 is further reduced, in this case including after phase separation, due to general electrostatic screening and competition for adsorption at the protein surface. Evidence of the competition between salt and buffer ions for protein surface binding is seen in Figure 3a; in the presence of competing buffer molecules, the influence of NaCl on KI8 LCST is reduced. We hypothesize that the “salting-in” effect of HEPES is at least somewhat dependent on the charged state of Lys side chains, as it has been previously demonstrated that buffer molecules can specifically adsorb to charged protein surfaces.^50^ To test this hypothesis and control for pH effects, we compared the effects of HEPES at pH 11.8 to HEPES pH 7.4 and deionized water pH 6.5 (Figure 5b). The *T*_t_ of KI8 in 10 mM HEPES pH = 11.8 (20 °C) is closer to the *T*_t_ of KI8 in deionized water (18 °C) than in HEPES pH = 7.4 (28 °C). Using amino acid p*K*_a_ values from Stryer, the theoretical change in the charge state of KI8 between pH 6.5 (deionized water) and pH 7.4 (HEPES) is from +10 to +9.7. If anything, one would expect a decrease in net charge to lead to a decrease in *T*_t_. However, since we observed an increase in transition temperature from 18 to 28 °C, it is implausible that pH alone can account for this effect.

The *T*_t_ observed for KI8 in CAPS pH 11.2 versus HEPES pH 11.8 increased from 16 to 20 °C. The theoretical change in the charge state of KI8 between these two pH values is from −1.4 to −1.8. One can hypothesize that the observed increase in *T*_t_ in this case may be due to an increase in charged surface area, a direct effect of HEPES, or a combination of the two. However, when compared to NaOH pH 12.9, which greatly decreases *T*_t_, the direction of the effect is the opposite. This further supports the claim that HEPES is directly increasing the solubility of KI8 and refutes an explanation that relies solely on pH effects. Given that the *T*_t_ is slightly elevated by HEPES even at high pH, HEPES may also weakly associate with the hydrophobic surfaces of KI8 to weaken the hydrophobic effect, a proposed mechanism for the salting-in effects observed with chaotropes even at low millimolar concentrations.^51^ Reported small molecule modulators of phase separation include bis-ANS and Congo red, both of which, like HEPES, contain negatively charged sulfonate groups (Figure 5c).^52^ Both HEPES and CAPS are zwitterionic buffers. If adsorption of buffer molecules is influencing KI8 interactions, a charged species might be expected to exert a greater effect than the zwitterionic form. According to the Henderson–Hasselbalch equation, at pH 7.4, HEPES will be 59.5% in its zwitterionic form and 41.5% in its anionic form. At pH 11.8, >99.99% of HEPES will be in its anionic form. If the anionic form of HEPES is predominantly responsible for adsorption to KI8, this could explain why a slight increase in *T*_t_ is still observed with HEPES at high pH even when KI8 is minimally charged. However, we do not rule out the possibility that the zwitterionic form of HEPES may also play a key role, given previous findings with other zwitterionic osmolytes such as glycine, betaine, and trimethylamine *N*-oxide, which have previously been shown to modulate ELP LCST behavior via multiple mechanisms.^53^

We performed temperature-ramp experiments on KI8 at various concentrations of HEPES, all at pH = 7.4 (Figure 5d). We observed an inverted U-shape dependence of *T*_t_ on HEPES concentration, similar to previously observed nonlinear effects of the chaotropic ions SCN^–^ and I^–^ on ELP and pNIPAM LCST behavior.^23,54,55^ HEPES at pH = 7.0 exhibits positive Jones–Dole *B* viscosity coefficient values, indicating a kosmotropic or “water-structuring” character.^45^ Our model system suggests that at low (<10 mM) concentrations, HEPES is predominantly acting by weakly associating with KI8. This weak association has multiple effects: it will decrease surface charge, increase conformational stability, and directly compete with protein–protein binding. While decreased surface charge should reduce charge–charge repulsion between KI8 monomers, the net effect appears to be increased solubility and stability of the monomeric form of KI8. At higher (≥100 mM) concentrations of HEPES as well as CAPS buffers, the buffers may act predominantly as classic kosmotropes, increasing the tendency of KI8 to undergo phase separation by disrupting hydrophobic hydration. Further investigation of these hypotheses using NMR to investigate direct ELP-buffer association and ITC to examine the corresponding entropic and enthalpic changes will be illuminating.

## Conclusions

Our DLS investigation of a polyelectrolyte ELP reports several key physical insights. First, in the course of attempting a straightforward, two-dimensional exploration of the behavior of the ELP KI8 in chemical space (pH vs ionic strength), we serendipitously created conditions in which KI8 appeared neither pH nor salt-responsive. Modulation of solution conditions could restore pH- and salt-responsiveness to the polymer as well as uncover novel cosolute influences. Although previous studies have quantitatively modeled the influence on LCST behavior of pH, salt concentration and identity, and polymer molecular weight, concentration, and composition, the vast potential of the experimental landscape makes it challenging to investigate and account for the complex interplay of these factors.^19−21,23,56−59^ Our findings underscore the importance of considering the limitations of existing quantitative models of ELP behavior and investigator intuition in bottom-up ELP design. Furthermore, we identify and characterize a novel influence of common biological buffer molecules on ELP LCST behavior. The buffer HEPES appeared to alter the LCST of KI8, increasing its solubility potentially through electrostatic interactions with lysine amino acids, rather than a direct pH effect. Overall, our findings are consistent with recent discussions of intrinsically disordered regions as inherent sensors of physicochemical changes.^60^ We hope that further systematic and wide-ranging investigations will follow. Key areas for exploration include the effect of cosolute molecules on rates of ELP assembly, the kinetics and hysteresis of the phase transition, how the sensitivity of an ELP to a given cosolute is encoded in its sequence, the influence of molecular crowding, and the thermodynamics of the various competing interactions present in the system. Along similar lines, we celebrate recent methodological advances in single live-cell imaging for the intracellular measurement of ELP *T*_t_.^61^ A deeper understanding of how cosolutes modulate ELP LCST behavior will facilitate their use as stimuli-responsive polymeric materials in various and dynamic environments and will be especially important to fully realize their promise within biological and industrial sensing applications.

## Supporting Information Available

Sequences of DNA and protein materials and results of SignalP cleavage site prediction (PDF)
