# Supplementary material for: Understanding the Phase Behavior of a Multistimuli-Responsive Elastin-like Polymer: Insights from Dynamic Light Scattering Analysis
Source: J Phys Chem B. 2024 Jun 3;128(23):5756–65. doi: 10.1021/acs.jpcb.4c00070 (PMC11181320; doi:10.1021/acs.jpcb.4c00070)

Title: Understanding the Phase Behavior of a Multi-Stimuli-Responsive Elastin-Like Polymer: Insights from Dynamic Light Scattering Analysis

Authorship

Peter C. Swanson[1], Galen P. Arnold[1], Carolyn E. Curley[1], Savannah C. Wakita[1], Jeffery D. Waters[1], Eva Rose M. Balog[1]*

Affiliation

[1]School of Mathematical and Physical Sciences, University of New England, Biddeford, Maine, USA, 04005.

*Corresponding author: Eva Rose M. Balog, ebalog@une.edu

Supporting Information

Contents:

Table S1. Sequences of DNA and protein materials.

Figure S1. Results of SignalP cleavage site prediction.

Table S1. Sequences of DNA and protein materials.

Feature name	DNA sequence	Translation	Purpose(s)
pelB leader	ATGAAATACCTATTGCCTACG<br>GCAGCCGCTGGATTGTTATTA<br>CTCGCAGCAAGCGGCGCGCA<br>TGCC	MKYLLPTAAAGL<br>LLLAASGAHA	Directs protein to periplasm (is removed upon translocation). Contains BsshII restriction site (GCGCGC) for cloning.
Linker	GCGGGCTGCGCAGGT	AGCAG	Functional group of Cys available as a chemical handle
ELP repeats – KI8	GTGCCGGGTAAAGGGGTGCC<br>TGGAATTGGCGTACCCGGCAT<br>CGGCGTGCCCGGTATTGGTGT<br>ACCGGGCATTGGTGTCCCTGG<br>CATCGGTGTTCCTGGCATTGG<br>TGTTCCGGGTATTGGTGTCCC<br>TGGTATTGGCGTGCCCGGCAA<br>AGGCGTGCCCGGTATTGGCGT<br>GCCTGGAATTGGAGTTCCGGG<br>TATTGGTGTTCCGGGTATTGG<br>TGTTCCAGGGATTGGTGTTCC<br>AGGTATCGGCGTACCGGGCA<br>TTGGCGTGCCCGGCATTGGCG<br>TCCCGGGTAAAGGCGTGCCTG<br>GCATTGGTGTTCCGGGTATCG<br>GCGTGCCCGGCATTGGCGTGC<br>CGGGCATTGGGGTGCCGGGT<br>ATTGGCGTTCCGGGGATTGGT<br>GTTCCGGGTATCGGCGTGCCG<br>GGGATTGGCGTGCCAGGGAA<br>GGGGGTACCGGGCATCGGCG<br>TTCCGGGAATTGGAGTCCCGG<br>GTATTGGGGTGCCGGGCATCG<br>GGGTCCCTGGCATTGGCGTGC<br>CGGGCATCGGGGTACCCGGA<br>ATCGGCGTGCCGGGTATTGGC<br>GTTCCGGGTAAAGGCGTGCC<br>AGGTATTGGCGTGCCAGGCAT<br>CGGTGTTCCGGGGATCGGTGT<br>ACCCGGCATCGGGGTGCCGG<br>GAATTGGAGTCCCGGGTATCG<br>GAGTACCGGGCATCGGCGTTC<br>CGGGCATCGGTGTCCCTGGCA<br>AGGGAGTGCCTGGCATTGGT<br>GTTCCAGGGATTGGGGTGCCA<br>GGAATCGGAGTCCCCGGGAT<br>TGGCGTTCCCGGTATCGGCGT<br>GCCGGGCATCGGCGTCCCGG<br>GCATTGGAGTACCGGGCATTG<br>GCGTTCCCGGCAAGGGCGTG<br>CCAGGTATTGGGGTTCCGGGT	VPGKGVPGIGVPG<br>IGVPGIGVPGIGVP<br>GIGVPGIGVPGIGV<br>PGIGVPGKGVPGI<br>GVPGIGVPGIGVP<br>GIGVPGIGVPGIGV<br>PGIGVPGIGVPGK<br>GVPGIGVPGIGVP<br>GIGVPGIGVPGIGV<br>PGIGVPGIGVPGIG<br>VPGKGVPGIGVPG<br>IGVPGIGVPGIGVP<br>GIGVPGIGVPGIGV<br>PGIGVPGKGVPGI<br>GVPGIGVPGIGVP<br>GIGVPGIGVPGIGV<br>PGIGVPGIGVPGK<br>GVPGIGVPGIGVP<br>GIGVPGIGVPGIGV<br>PGIGVPGIGVPGIG<br>VPGKGVPGIGVPG<br>IGVPGIGVPGIGVP<br>GIGVPGIGVPGIGV<br>PGIGVPGKGVPGI<br>GVPGIGVPGIGVP<br>GIGVPGIGVPGIGV<br>PGIGVPGIGVPGK<br>GVPGIGVPGIGVP<br>GIGVPGIGVPGIGV<br>PGIGVPGIGVPGIG<br>VPGKGVPGIGVPG<br>IGVPGIGVPGIGVP<br>GIGVPGIGVPGIGV<br>PGIGVP	Stimuli-responsive assembly behavior

	ATTGGAGTACCGGGCATCGG<br>GGTTCCGGGAATTGGAGTACC<br>AGGCATCGGTGTGCCAGGTAT<br>CGGAGTGCCGGGCATCGGCG<br>TTCCAGGCATTGGAGTCCCGG<br>GGAAAGGAGTACCGGGGATC<br>GGCGTTCCCGGCATCGGGGTA<br>CCTGGTATCGGTGTTCCGGGC<br>ATTGGGGTGCCGGGTATTGGC<br>GTGCCTGGTATCGGGGTACCG<br>GGAATTGGGGTGCCGGGTATT<br>GGGGTCCCTGGCAAGGGAGT<br>GCCGGGGATTGGTGTCCCCGG<br>CATTGGAGTGCCGGGAATTG<br>GTGTTCCGGGCATTGGCGTTC<br>CCGGTATTGGTGTGCCTGGTA<br>TTGGTGTTCCCGGGATTGGTG<br>TCCCGGGCATTGGGGTCCCGG<br>GCAAAGGCGTACCAGGTATC<br>GGTGTCCCCGGCATCGGTGTC<br>CCGGGTATCGGCGTGCCAGG<br>CATCGGGGTGCCGGGCATTG<br>GCGTTCCTGGTATTGGCGTTC<br>CAGGCATTGGCGTGCCAGGG<br>ATTGGAGTGCCG		
ELP repeats – I90	GTTCCGGGGATTGGTGTTCCT<br>GGCATCGGCGTTCCGGGCATT<br>GGCGTTCCGGGGATCGGCGTT<br>CCGGGCATTGGAGTGCCGGG<br>CATCGGTGTGCCTGGAATTGG<br>TGTTCCGGGTATCGGCGTTCC<br>AGGTATCGGTGTACCAGGGA<br>TTGGCGTCCCGGGTATCGGCG<br>TTCCAGGTATCGGCGTGCCGG<br>GCATTGGTGTACCGGGCATTG<br>GAGTTCCGGGCATTGGTGTCC<br>CGGGTATTGGCGTCCCAGGTA<br>TCGGCGTCCCTGGGATCGGCG<br>TTCCTGGTATCGGTGTGCCAG<br>GCATCGGTGTGCCTGGCATTG<br>GCGTCCCAGGCATCGGTGTGC<br>CGGGAATTGGGGTGCCAGGG<br>ATCGGCGTTCCTGGCATTGGT<br>GTACCGGGAATCGGTGTTCCT<br>GGGATCGGTGTACCGGGTATC<br>GGGGTGCCAGGCATTGGTGTT<br>CCCGGTATCGGCGTACCGGGC<br>ATCGGCGTACCGGGTATCGGT<br>GTGCCAGGTATCGGCGTGCCA<br>GGTATCGGCGTGCCAGGCATT<br>GGTGTCCCGGGTATTGGTGTC<br>CCAGGCATCGGCGTCCCGGGT<br>ATCGGTGTTCCGGGTATTGGT	VPGIGVPGIGVPGI<br>GVPGIGVPGIGVP<br>GIGVPGIGVPGIGV<br>PGIGVPGIGVPGIG<br>VPGIGVPGIGVPGI<br>GVPGIGVPGIGVP<br>GIGVPGIGVPGIGV<br>PGIGVPGIGVPGIG<br>VPGIGVPGIGVPGI<br>GVPGIGVPGIGVP<br>GIGVPGIGVPGIGV<br>PGIGVPGIGVPGIG<br>VPGIGVPGIGVPGI<br>GVPGIGVPGIGVP<br>GIGVPGIGVPGIGV<br>PGIGVPGIGVPGIG<br>VPGIGVPGIGVPGI<br>GVPGIGVPGIGVP<br>GIGVPGIGVPGIGV<br>PGIGVPGIGVPGIG<br>VPGIGVPGIGVPGI<br>GVPGIGVPGIGVP<br>GIGVPGIGVPGIGV<br>PGIGVPGIGVPGIG<br>VPGIGVPGIGVPGI<br>GVPGIGVPGIGVP<br>GIGVPGIGVPGIGV<br>PGIGVPGIGVPGIG	Stimuli-responsive assembly behavior

	GTTCCCGGTATTGGGGTCCCA<br>GGGATTGGGGTGCCAGGTAT<br>CGGTGTTCCGGGTATTGGAGT<br>GCCCGGGATCGGCGTCCCAG<br>GTATTGGTGTGCCTGGAATCG<br>GGGTGCCGGGTATCGGGGTC<br>CCGGGGATCGGCGTGCCAGG<br>TATTGGTGTTCCTGGCATCGG<br>CGTGCCGGGCATCGGGGTCCC<br>AGGGATCGGCGTCCCTGGCAT<br>TGGCGTTCCAGGTATCGGCGT<br>ACCTGGCATTGGCGTGCCGGG<br>CATTGGAGTGCCAGGTATCGG<br>GGTGCCCGGGATCGGCGTAC<br>CAGGCATCGGAGTGCCAGGC<br>ATTGGCGTGCCGGGCATCGGC<br>GTTCCGGGAATCGGAGTACC<br>AGGCATTGGAGTGCCGGGCA<br>TCGGCGTTCCGGGGATCGGG<br>GTGCCGGGGATTGGCGTACCC<br>GGTATTGGCGTACCGGGTATC<br>GGTGTTCCCGGTATTGGCGTT<br>CCGGGCATCGGTGTTCCAGGC<br>ATTGGCGTTCCAGGTATCGGT<br>GTACCGGGAATTGGTGTCCCT<br>GGCATCGGTGTACCGGGCATT<br>GGTGTCCCGGGCATTGGTGTA<br>CCGGGTATCGGCGTCCCAGGC<br>ATCGGCGTACCAGGTATCGGT<br>GTGCCGGGTATCGGCGTGCCA<br>GGCATTGGTGTTCCAGGTATC<br>GGTGTGCCGGGGATTGGGGT<br>GCCGGGGATTGGTGTACCTGG<br>TATTGGTGTTCCGGGCATTGG<br>CGTTCCAGGGATCGGTGTGCC<br>AGGTATCGGCGTTCCGGGGAT<br>CGGCGTCCCAGGCATTGGAGT<br>GCCC	VPGIGVPGIGVPGI<br>GVPGIGVPGIGVP<br>GIGVPGIGVPGIGV<br>PGIGVPGIGVPGIG<br>VPGIGVPGIGVP	
Trp tag	GCTAGCTGGTGA	ASW*	Allows detection using absorbance at 280 nm; potential chemical handle. Contains NheI restriction site for cloning (GCTAGC) and stop codon (TGA).

Figure S1. Results of SignalP cleavage site prediction.

Cleavage site between pos. 22 and 23. Probability 0.969145

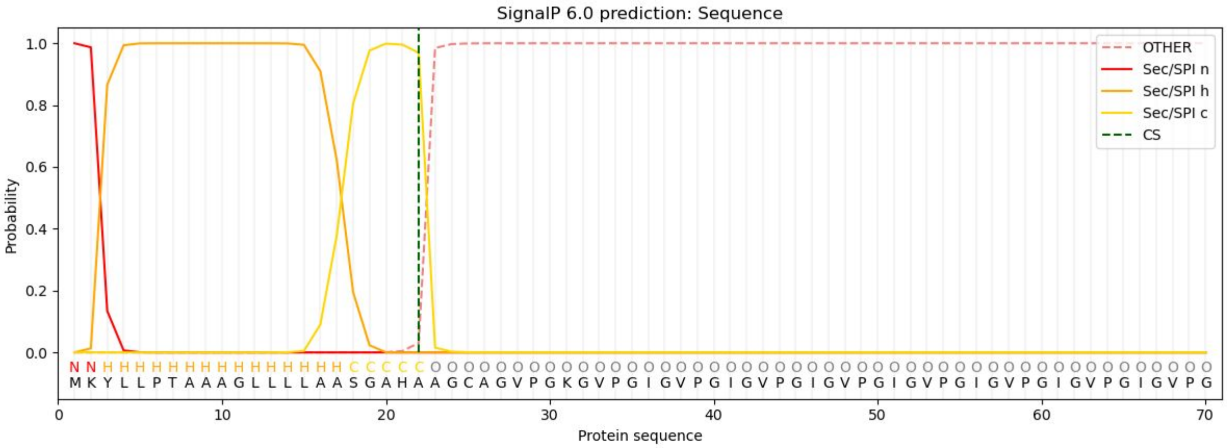

Supplement: Supplementary file 1 — jp4c00070_si_001.pdf [file jp4c00070_si_001.pdf]
